# Support-vector classification of low-dose nitrous oxide administration with multi-channel EEG power spectra

**DOI:** 10.1007/s10877-023-01054-w

**Published:** 2023-07-13

**Authors:** Xavier C. E. Vrijdag, Luke E. Hallum, Emma I. Tonks, Hanna van Waart, Simon J. Mitchell, Jamie W. Sleigh

**Affiliations:** 1 Department of Anaesthesiology, School of Medicine, University of Auckland, Private Bag 92019, Auckland, 1142 New Zealand; 2 Department of Mechanical and Mechatronics Engineering, University of Auckland, Auckland, 1142 New Zealand; 3 Department of Anaesthesia, Auckland City Hospital, Auckland, 1023 New Zealand; 4 Department of Anaesthesia, Waikato Hospital, Hamilton, 3240 New Zealand

**Keywords:** Nitrous oxide, EEG, Support vector machine, Machine learning, Monitoring, Anaesthesia

## Abstract

Support-vector machines (SVMs) can potentially improve patient monitoring during nitrous oxide anaesthesia. By elucidating the effects of low-dose nitrous oxide on the power spectra of multi-channel EEG recordings, we quantified the degree to which these effects generalise across participants. In this single-blind, cross-over study, 32-channel EEG was recorded from 12 healthy participants exposed to 0, 20, 30 and 40% end-tidal nitrous oxide. Features of the delta-, theta-, alpha- and beta-band power were used within a 12-fold, participant-wise cross-validation framework to train and test two SVMs: (1) binary SVM classifying EEG during 0 or 40% exposure (chance = 50%); (2) multi-class SVM classifying EEG during 0, 20, 30 or 40% exposure (chance = 25%). Both the binary (accuracy 92%) and the multi-class (accuracy 52%) SVMs classified EEG recordings at rates significantly better than chance (p < 0.001 and p = 0.01, respectively). To determine the relative importance of frequency band features for classification accuracy, we systematically removed features before re-training and re-testing the SVMs. This showed the relative importance of decreased delta power and the frontal region. SVM classification identified that the most important effects of nitrous oxide were found in the delta band in the frontal electrodes that was consistent between participants. Furthermore, support-vector classification of nitrous oxide dosage is a promising method that might be used to improve patient monitoring during nitrous oxide anaesthesia.

**Supplementary Information:**

The online version contains supplementary material available at 10.1007/s10877-023-01054-w.

## Introduction

Nitrous oxide is a weak anaesthetic gas with hypnotic and analgesic properties, which has strong dissociative effects [1]. It is often used in isolation to induce a relaxed state and provide analgesia in dentistry, obstetrics, or acute trauma [2]. In the operating theatre, nitrous oxide may be combined with other anaesthetic vapours to achieve general anaesthesia [3].

Depth of anaesthesia monitoring is commonly used during surgery for patient safety [4]. Monitoring the anaesthetic depth reduces the risk of patient awareness intraoperatively, and also reduces recovery time, as it mitigates anaesthetic overdosing [5]. These monitors are based on characteristic alterations in electroencephalogram (EEG) recordings induced by anaesthetic drugs. Gamma-amino-butyric-acid (GABA)-ergic drugs are most commonly used for inducing unconsciousness. Therefore, most anaesthetic depth monitors are calibrated for GABA-ergic drugs, like propofol [4], but are insensitive to the effects of nitrous oxide [6–9]. This insensitivity is likely underpinned by different electrophysiological effects arising from targeting different receptors, as nitrous oxide primarily binds to the N-methyl-D-aspartate (NMDA) receptor [1].

Studies of alterations in the EEG power spectrum during nitrous oxide exposure have produced mixed results. An increase in power in high-frequency bands (beta and gamma) is often reported, with a relative decrease in power in the lower frequency bands (delta and alpha) [10]. This power reduction [11] is localised in the frontal region [12, 13] and most pronounced at higher exposures (i.e., 40 to 60%) [12, 14, 15]. Nitrous oxide also reduces functional connectivity in parietal (at 60% nitrous oxide) and frontal (16 to 30% nitrous oxide) regions [13, 16]. Our own group reported a reduction in temporal complexity in the central region that tracked cognitive impairment caused by nitrous oxide inhalation [17].

Machine-learning classification can analyse EEG recordings and is increasingly used in medicine and biology for behavioural and mental state monitoring [18–20]. To do so, labelled EEG recordings are represented in a derived feature space. This representation can be used, first, to infer which features are diagnostic (i.e., which features discriminate EEG recordings of one category from another) and, second, to assess the generalizability of diagnostic features across participants. Support-vector machines (SVMs) are one such approach to classification. SVMs fit a discriminant function to the derived feature space so as to maximise the margin separating EEG recordings from two categories [21]. SVMs offer several advantages to other classification approaches: they often generalise with good accuracy where high-dimensional data are concerned, and SVMs often yield readily interpretable results (i.e., the determination of the diagnostic features) [22], making them algorithmically explainable.

SVM performance relies heavily on the chosen EEG features. A common way of quantifying EEG is by calculating the power in pre-defined frequency bands (delta, theta, alpha and beta) [10]. These features are commonly used in EEG analysis for level of anaesthesia classification as they are easy to calculate, and have a physiological meaning as they are known to be related to certain brain states and to be driven by specific brain regions [23–27], making them biologically explainable. Besides frequency, entropy is also commonly used as a feature input for SVM modelling [24, 26, 28–30]. SVM classification of the depth of anaesthesia for all kinds of anaesthetics (excluding nitrous oxide) has been compared with other machine-learning algorithms, like random forest classification, regression, and artificial neural networks, with mixed results [26, 27, 29]. For this reason we will only focus on SVM analysis and will investigate the features selected by the SVMs to help explain the SVM algorithmically and biologically.

This study aimed to analyse previously recorded EEG [17] with SVMs to investigate their use to improve patient monitoring during nitrous oxide anaesthesia. By classifying the effects of low-dose nitrous oxide on the power spectra of multi-channel EEG recordings, we quantified the degree to which these effects generalise across participants.

## Materials and methods

### Trial design and participants

This secondary analysis study is based on multidose data collected in a single-blind, cross-over trial conducted at the Waikato Clinical School, University of Auckland, in July and August 2018. The original study aim was to expose healthy volunteers to nitrous oxide to develop a quantitative EEG analysis algorithm to detect cognitive impairment in scuba divers. This study was performed in line with the principles of the Declaration of Helsinki. The study protocol was approved by the Health and Disability Ethics Committee, Auckland, New Zealand (16/NTA/93), and was registered with the Australian New Zealand Clinical Trial Registry (U1111-1181-9722, RRID:SCR_002967).

Recruitment and inclusion and exclusion criteria are described in more detail elsewhere [17]. In summary, participants were healthy, certified divers aged between 18 and 60 years. Candidate participants currently using recreational drugs, tobacco, psychoactive medication, excessive alcohol, or over five caffeine-containing beverages a day were excluded. Participants had at least 6 h of sleep, abstained from any caffeinated drink on the day, fasted for 4 h and refrained from alcohol for 24 h prior. All participants provided written informed consent.

### Experimental procedures

Twelve participants visited the facility once. Each experimental session began with a baseline measurement without nitrous oxide, followed by titrated gas mixtures to produce 20, 30 and 40% end-tidal nitrous oxide (in random order) using an anaesthetic circuit. There were 20-min breaks between the nitrous oxide exposures. Each exposure started with a 3–5 min wash-in until stable end-tidal nitrous oxide levels were achieved. The EEG was recorded directly after the wash-in period and at the end of the 10-min exposure with one minute eyes-open and one minute eyes-closed.

The EEG was recorded at 1024 Hz using a portable active electrode 32-channel system (ActiveTwo, BioSemi, Amsterdam, the Netherlands) divided over the scalp based on the international 10–10 system [31] with two additional electrooculogram electrodes placed under the eyes. The offset (impedance equivalent for active systems) was maintained below 25 µV with gelling (SignaGel, Parker Laboratories, Fairfield, NJ, USA).

### EEG pre-processing and feature extraction

EEG pre-processing removed eye movement, muscle and noise artefacts using the Fieldtrip toolbox (version c6d58e9 RRID:SCR_004849) [32]. The data for the eyes-open condition at the end of each exposure was segmented from the continuous recording, re-referenced to the average, de-meaned, de-trended, and resampled to 256 Hz. Line (50 Hz, including higher harmonic) and low-frequency noise (< 1 Hz) were filtered out. Next, independent component analysis was used to filter noise components like eye blinks, high-frequency noise, non-physiological noise, and bad channels. The data were manually inspected for remaining artefacts. Whole two-second epochs containing artefacts were discarded (Matlab code is available at [17]).

Spectral power was estimated with a multitaper Fourier transformation implemented in the Fieldtrip toolbox using a Hanning window from 1 to 30 Hz with 1 Hz increments. The average power per frequency band was calculated for: delta (1–4 Hz), theta (4–8 Hz), alpha (8–14 Hz) and beta (14–30 Hz) for each exposure and channel as 128 features (4 frequency bands × 32 channels) for the SVM classification.

### Initial exploratory analysis

At an early stage of our analysis, we wondered whether a traditional approach to classification via linear dimensionality reduction would provide insight into the nature of the recordings, specifically, whether the high-dimensional feature space (4 frequency bands × 32 channels) comprised a signal enabling classification. We adapted the approach described by Jamieson et al. [33], to explore whether a traditional approach to classification via linear dimensionality reduction would provide insight into the nature of the recordings. We applied principal component analysis to the high-dimensional representation (4 frequency bands × 32 channels) of EEG recordings during 0 and 40% nitrous oxide exposure. This low-dimensional representation of recordings (i.e., principal component scores) was used as input to a simple Fisher’s linear discriminant analysis (i.e., binary classification). We used 12-fold, leave-one-participant-out cross-validation for training and testing (on fold 1, we trained the linear discriminant analysis using data recorded from participants 1 through 11, and then tested the trained linear discriminant analysis using data recorded from participant 12; on fold 2, training used participants 1 through 10 and 12, and testing used participant 11; and so on). To evaluate the performance of the linear discriminant analysis, a confusion matrix was calculated for each leave-one-participant-out fold; overall accuracy was calculated by averaging accuracy across folds. The theoretical baseline of a binary classifier accuracy is 50% correct; we estimated an empirical baseline for our linear discriminant analysis using a shuffle test [34]. To do so, on each of 100 iterations, we repeated the above-described procedure, but first shuffled labels (“0% exposure” or “40% exposure”) on training data (not testing data). The empirical baseline was computed by averaging overall accuracy across iterations.

### SVM classification

EEG recordings during 0 or 40% exposure were classified using an SVM (i.e., binary classification) and 12-fold, leave-one-participant-out cross-validation. We used a linear SVM because preliminary testing indicated a linear SVM’s performance was comparable to that of SVMs involving higher-order polynomial kernels or a Gaussian kernel. Other hyper parameters (box constraint, kernel scale and standardization) appeared to have no influence on model performance, and were left to the default. To evaluate the performance of the SVM, a confusion matrix was calculated for each leave-one-participant-out fold; overall accuracy was calculated by averaging accuracy across folds. We estimated an empirical baseline for the SVM using a shuffle test, similar to the method described in the initial exploratory analysis [34]. We also classified EEG recordings during 0, 20, 30 or 40% nitrous oxide exposure using a “one-versus-one” multi-class SVM model.

To gain a deeper understanding of which EEG features enabled classification, we repeated the binary and multi-class SVM analyses using "lesioned" datasets. Each lesioned dataset comprised only the features of one frequency band (e.g., alpha), or only the features of three frequency bands (e.g., delta, theta, and beta), thus quantifying the role of an individual frequency band (e.g., alpha) in classification performance. Similarly, EEG channels were divided into four regions (frontal, lateral, central and occipital). These regions were used to perform another lesioning scheme to explore the regional variation. The effects of "lesioning" on performance were visualised using violin plots [35].

### Statistical analysis

Model performance is denoted as mean accuracy with a binomial 95%-confidence interval. We compared model performance (either binary or multi-class) of the complete model with the empirical baseline using a two-tailed, paired Wilcoxon signed test, as data were not normally distributed (Shapiro–Wilk test). If significant, we performed a further analysis with the complete model, the empirical baseline model and the lesioned dataset models (e.g., delta, theta, alpha, and beta, and data from three frequency bands) using a Friedman's test for inequivalence between the models. For post-hoc analysis, we used a Nemenyi test for pairwise comparisons of all models, as suggested by Demsar [36, 37]. Differences were regarded as significant at p < 0.05. All analyses were performed using Matlab version 2022a (Mathworks, Natick, MA, USA, RRID:SCR_001622) and are available in Online Appendix 1.

## Results

In pre-processing, we removed on average 1.7 of every 30 two-second-long EEG samples due to artefacts (5.7%; average across participants). Most samples were removed due to muscle-related artefacts.

### Initial exploratory analysis

**Fig. 1:**
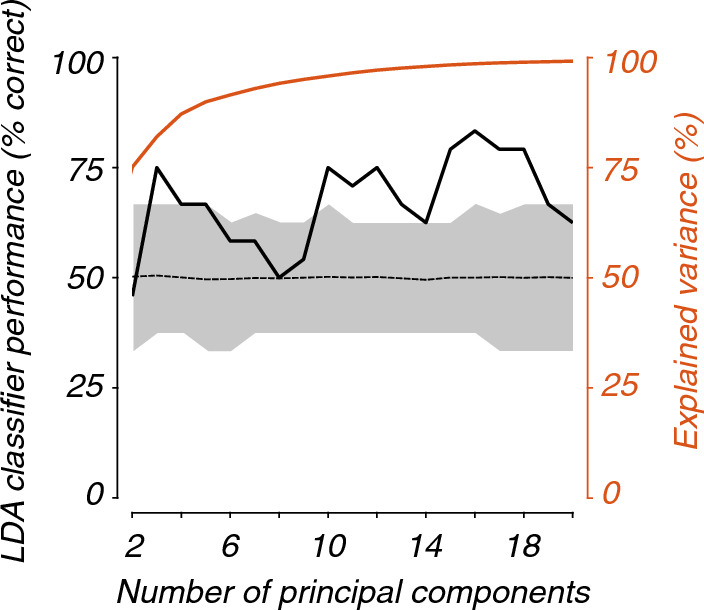
Initial exploratory analysis using principal component analysis and linear discriminant analysis showed that EEG recordings contained a signal-enabling classification of 0% vs 40% nitrous oxide exposure. The principal component analysis reduced the dimensionality of the data set, with explained variance in red. After dimensionality reduction with principal component analysis, we used leave-one-participant-out cross-validation to assess a simple linear discriminant analysis classifier, with classifier performance in black. A shuffle test indicated that the empirical baseline was approximately 50% (dashed curve), with a 95% confidence interval (shaded) between 33 and 66%

The initial exploratory analysis indicated the presence of a signal for classification (Fig. 1). As expected, explained variance (red curve) increased with the number of principal components such that 95% and 99% of the variance was explained by 9 and 19 components. Classifier performance increased with the number of components (black curve), e.g., using 16 components enabled classifier performance of 83.3% correct. This performance was significantly better than chance.

### Binary SVM model

**Fig. 2:**
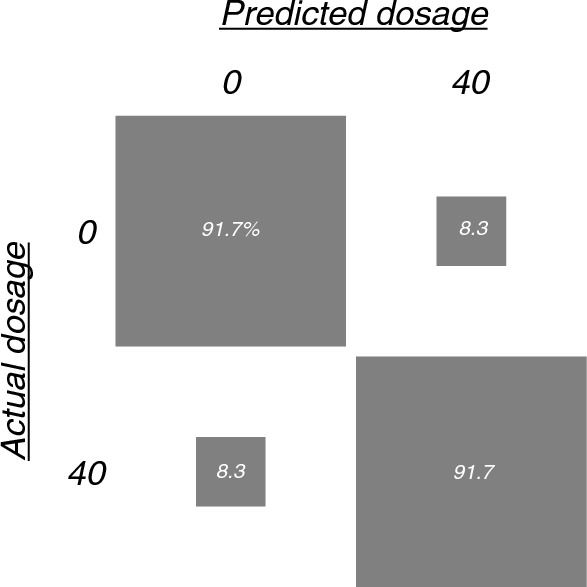
Confusion matrix showing classification of EEG recordings during 0 or 40% nitrous oxide exposure. To assess SVMs generalizability, 12-fold participant-wise cross-validation was used

The binary SVM model achieved an average classification accuracy of 92% (95%CI 73% to 99%) compared to the chance-level accuracy calculated with the shuffle test (49%) (p < 0.001). The confusion matrix shows the predicted versus true exposures (Fig. 2).

**Fig. 3:**
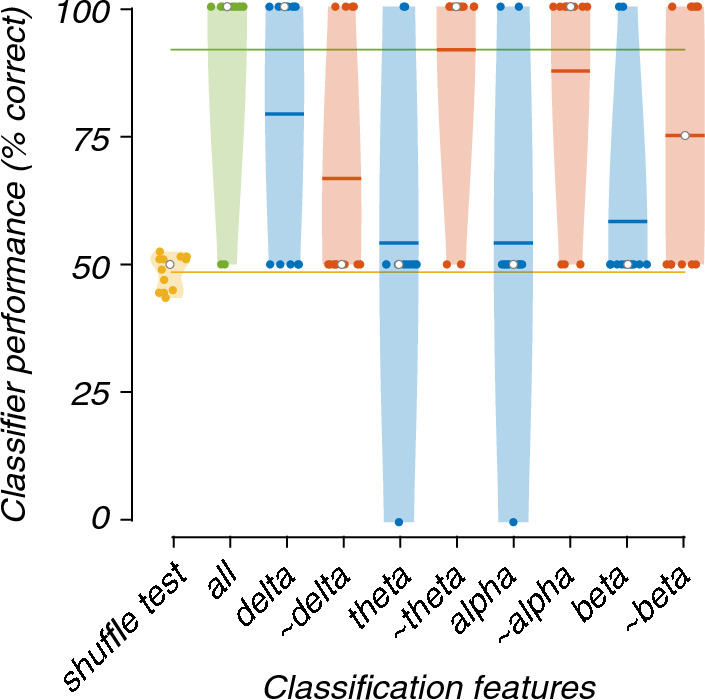
Violin plots showing the contribution of each frequency band to the classification of EEG recordings during 0 or 40% nitrous oxide exposure. The green plot ("all") shows classifier performance when all bands (delta, theta, alpha, and beta) were used in the training and testing of the classifier. The yellow plot ("shuffle test") shows the empirical baseline. The leftmost blue plot ("delta") shows the classifier's performance when only delta-band activity was used in classifier training and testing; the leftmost red plot ("~ delta") shows performance when only theta-, alpha-, and beta-band activity was used. Similarly, for the other blue and red plots. Filled symbols show accuracy on each fold of the 12-fold participant-wise cross-validation. Open symbols mark medians, and horizontal lines mark means

**Fig. 4:**
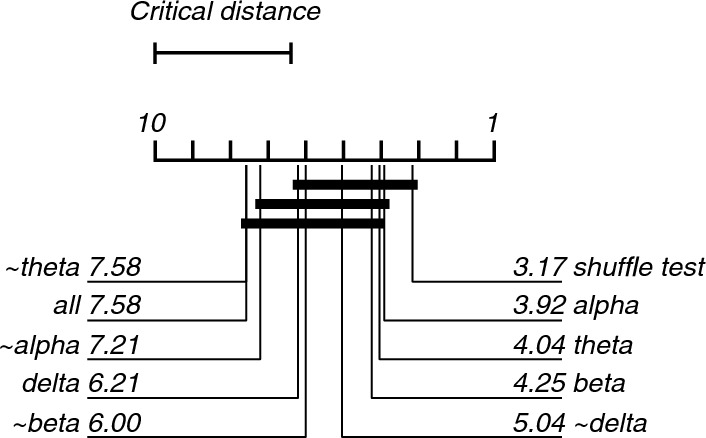
Critical difference diagram displaying statistical significance between accuracies of the models trained and tested during the classification of EEG recordings during the 0 and 40% nitrous oxide exposures. Model numbers are mean ranks, where low ranks indicate that a model has a lower accuracy than its competitors with higher ranks. Critical difference of 3.6. Two or more models are connected with horizontal bars if there is no statistically significant difference. This analysis shows statistically significant difference between the 'all', '~ theta' and '~ alpha' models compared with the shuffle test model

As expected, all frequency band lesioned models performed worse than the complete model (i.e., the model trained and tested on all frequency-band features; Fig. 3). However, the model containing only the delta-band features showed reasonably good accuracy (79%) compared to the complete model. In contrast, the models trained and tested using only theta-, alpha- or beta-band features showed much-reduced accuracy (54, 54 and 58%, respectively). The Friedman's test showed a statistically significant difference between the performance of models (X^2^(9) = 41.05, p < 0.001). Post-hoc analysis revealed that the models trained and tested, which did not contain the alpha- or theta-band (~ alpha and ~ theta) features, were significantly better than the chance-level accuracy (Fig. 4).

**Fig. 5:**
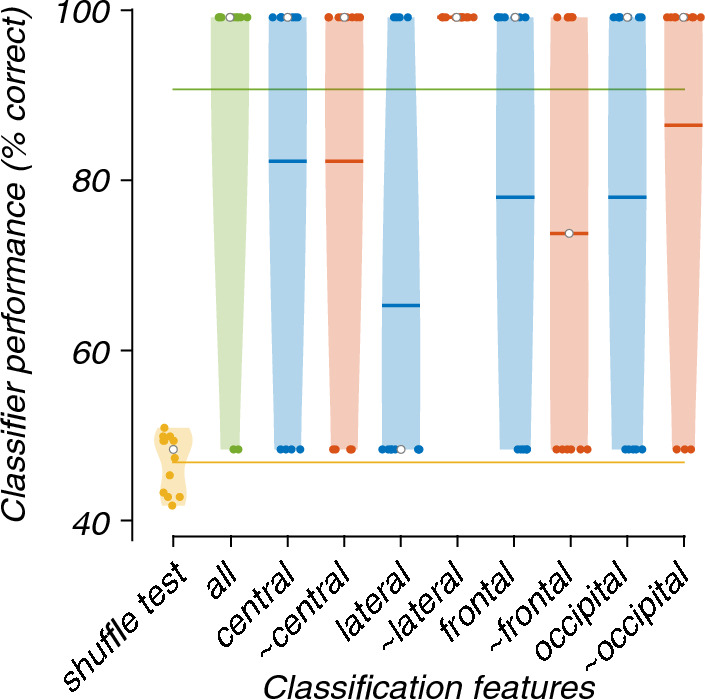
Violin plots showing the contribution of each region to the classification of EEG recordings during 0 or 40% exposure. All graphical conventions are as in Fig. 3

**Fig. 6:**
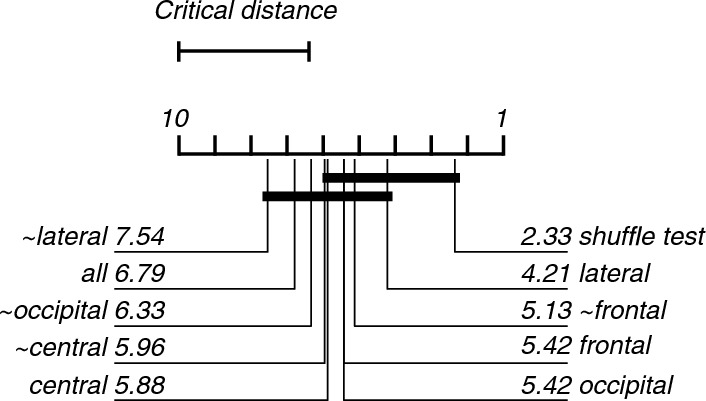
Critical difference diagram displaying the statistical significance between the accuracies of the models trained and tested during the classification of EEG recordings during the 0 and 40% nitrous oxide exposures. All graphical conventions are as in Fig. 4

In most cases, the lesioning of electrode regions only marginally reduced the accuracy of the models. The exceptions are the model containing only the lateral electrode features (67%) and the model not containing the frontal electrode features (~ frontal; 75%; Fig. 5). The Friedman's test showed a significant difference between the performance of models (X^2^(9) = 37.47, p < 0.001). Post-hoc analysis revealed that the models trained and tested, which did not contain the lateral or occipital electrode (~ lateral and ~ occipital) features, were significantly better than the chance-level accuracy (Fig. 6).

### Multi-class SVM model

**Fig. 7:**
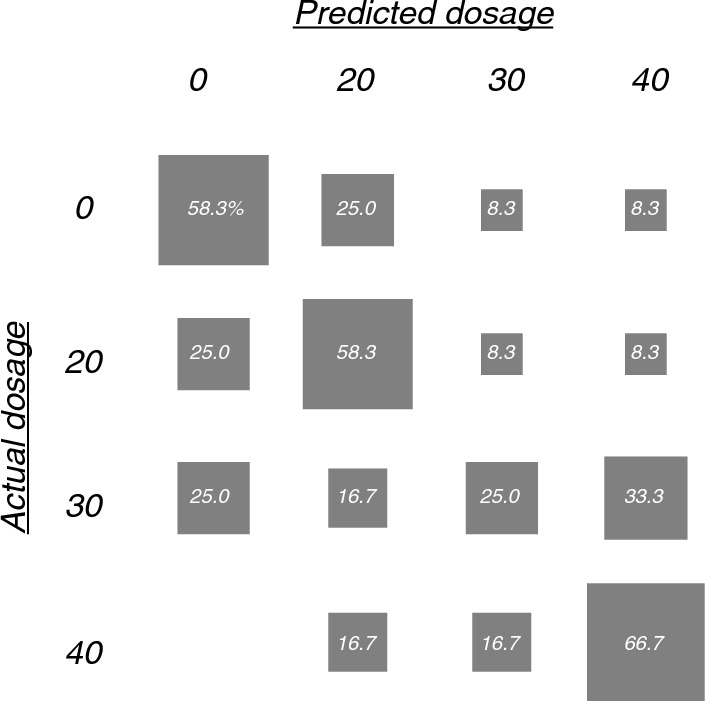
Confusion matrix showing classification of EEG recordings during 0, 20, 30 or 40% nitrous oxide exposure. To assess SVMs generalizability, 12-fold participant-wise cross-validation was used. The size of the boxes indicates the level (in)correctness

The multi-class SVM model was significantly better, with an average classification accuracy of 52% (95%-CI 37% to 67%), compared to chance-level accuracy calculated with the shuffle test (26%, p = 0.01). The confusion matrix shows the predicted versus true exposures. Sensitivity (3/12 = 25%) was lowest for the 30% exposure; which the model tended to confuse with the 0 and 40% exposures (Fig. 7).

**Fig. 8:**
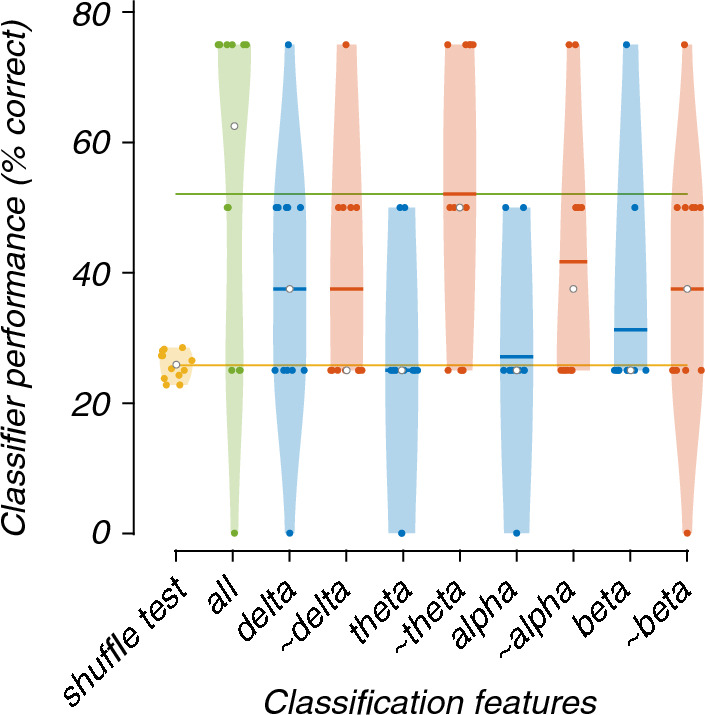
Violin plots showing the contribution of each frequency band to the classification of EEG recordings during 0, 20, 30, or 40% exposure. All graphical conventions are as in Fig. 3

**Fig. 9:**
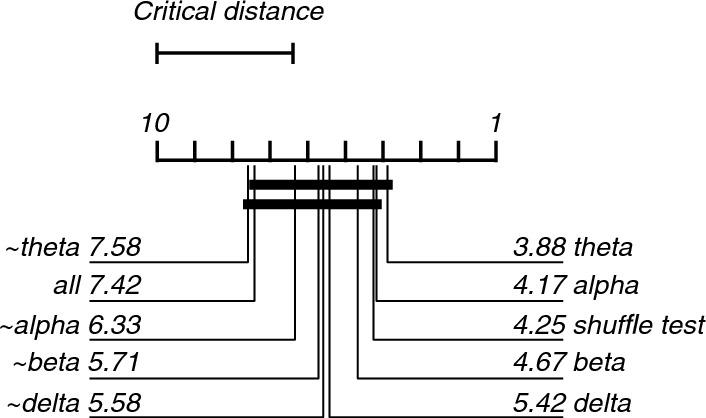
Critical difference diagram displaying the statistical significance between the accuracies of the models trained and tested during the classification of EEG recordings during the 0, 20, 30 and 40% nitrous oxide exposures. All graphical conventions are as in Fig. 4

As expected, all frequency band lesioned multi-class models performed worse than the complete model (Fig. 8). The models trained and tested using only the theta- (25%) or alpha-band (27%) features performed worse than the complete model. The fold with 0% accuracy of the complete model is of the same participant as the 0% accuracy fold of the delta-only lesioning. The Friedman’s test showed a statistically significant difference between the performance of models (X^2^(9) = 25.43, p = 0.003). Post-hoc analysis revealed that only the model which did not contain the theta-band features (~ theta), was significantly better than chance-level accuracy based on the shuffle test (Fig. 9).

**Fig. 10:**
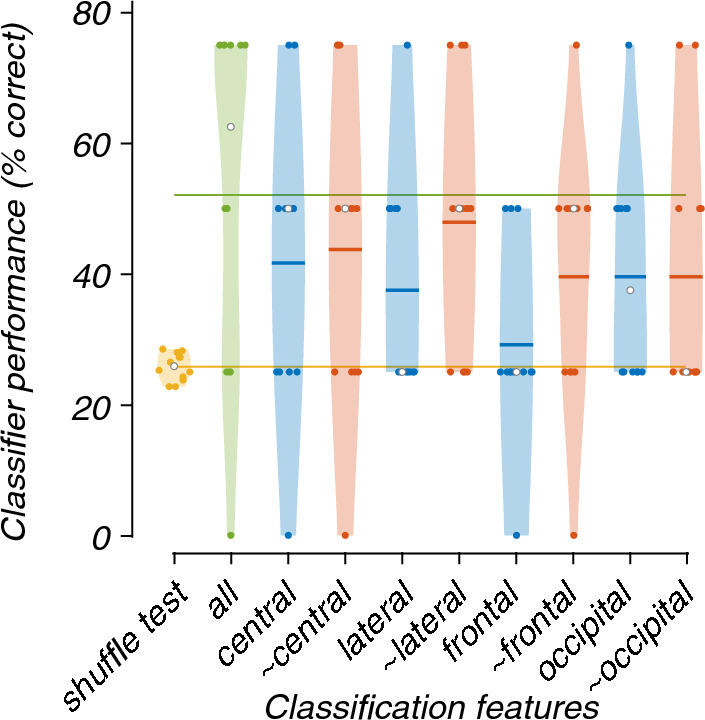
Violin plots showing the contribution of each region to the classification of EEG recordings during 0, 20, 30, or 40% exposure. All graphical conventions are as in Fig. 3

**Fig. 11:**
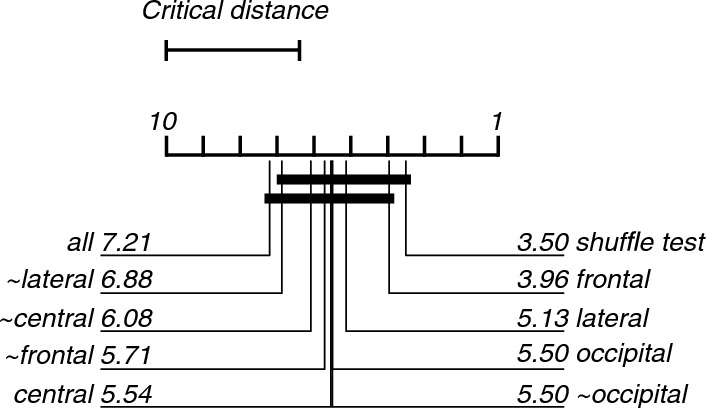
Critical difference diagram displaying the statistical significance between the accuracies of the models trained and tested during the classification of EEG recordings during the 0, 20, 30 and 40% nitrous oxide exposures. All graphical conventions are as in Fig. 4

The regional lesioning of features only marginally reduced the accuracy, except for the model only containing the frontal electrode features (Fig. 10). Friedman's test and post-hoc analysis did not show statistically significant differences between the lesioned models (Fig. 11).

## Discussion

This study aimed to investigate the use of SVMs to improve patient monitoring during nitrous oxide anaesthesia. By classifying whether low-dose nitrous oxide affected certain frequency bands of the power spectra of multi-channel EEG recordings, and whether these effects generalised across participants. We evaluated two SVMs: a binary SVM and a multi-class SVM. Both identified the same frequency bands and electrode regions. The binary model, which classified 0 and 40% nitrous oxide exposures, identified that the absence of the theta- or alpha-band or lateral or occipital electrodes did not significantly change the classification accuracy. The multi-class model classified 0, 20, 30 and 40% nitrous oxide exposure and found similar results for the theta band, i.e., the most important effects of nitrous oxide were found in both extremes of the frequency range (decreased power in the delta band and increased power in the beta band) in the frontal and central electrodes.

In the initial exploratory analyses, we showed that the principal component analysis with linear discriminant analysis method proved to have a viable signal in the dataset that could be classified. The subsequent SVM parameter optimisation showed that the linear model was comparable to the higher polynomial kernels. Given that the principal component analysis with linear discriminant analysis is also a linear method, it is not surprising that the SVM linear model had a similar performance.

The performance accuracy of the binary (92%) and multi-class (52%) SVMs is slightly lower than the accuracies of SVM models in other anaesthetics [24–30]. This could be because nitrous oxide produces less pronounced EEG effects. However, a more likely explanation is that most other studies classified over a larger dosage range, up to dosages that caused unconsciousness [23, 24, 28–30]. Our study only used low-dose nitrous oxide, enough to produce analgesia but without causing unconsciousness.

### Frequency power lesioning

The frequency power analysis performed in the original study found a power increase in the beta band and a relative decrease in the delta band, most prominent in the frontal region and most visible in the 20% and 40% exposures [17]. These changes might help explain the frequency band and electrode regional lesioning results, which found that delta and beta in the frontal and central regions supported classification.

The alterations in the power spectra of EEG recordings are inconsistent with some previous descriptions of changes in EEG caused by increased dosage of nitrous oxide [10, 13] but consistent with others [11, 12, 38]. These differences might be due to variations in dosage, administration method, and exposure time or differences in the pre-processing of the EEG signals. The studies cited above have contributed to the debate about the importance of alpha-band power to characterise the effects of nitrous oxide. Our study has shown that features based on the alpha frequency band do not contribute to classification accuracy, but did illustrate the importance of the delta band.

### Binary versus multi-class model

The binary and multi-class analysis indicated that the theta and alpha frequency bands are of no interest in distinguishing nitrous oxide dosage. Moreover, the multi-class model confirmed the unimportance of the theta-band. The difference between binary and multi-class analyses could be due to the increased complexity of the multi-class model because it needs to classify smaller differences. Compared to the binary model, which only had to classify between 0 and 40% nitrous oxide.

### Strengths and limitations

Our study had two strengths in particular. First, the dataset contained four nearby levels of low-dose nitrous oxide, which is a challenging task to assign to any classification model. Secondly, the SVM machine-learning algorithm we chose is computationally fast and robust with high-dimensional data, making it easy to implement into a clinical monitor.

Our study also had some limitations. First, we recruited only 12 healthy participants, which may limit the external validity of our results (i.e., the ability of these results to generalise to other, larger cohorts with different health profiles). However, we note the good internal validity of our models; that is, our models performed well within a 12-fold, leave-one-participant-out cross-validation framework. Additional data and analysis might further improve the algorithm and confirm these findings. Second, the data analysis focused only on SVM, as it is a robust method with the benefit of minimising overfitting on the small available dataset. Furthermore, only power spectral features were used to train the SVM, as they are biologically explainable. Future research could extend into other machine learning algorithms and quantitative EEG metrics like entropy as features, as they could provide novel insights and be compared against each other. Third, we recorded EEG using 32 electrodes covering the whole brain, and recordings on all electrodes were incorporated into the model as a feature. To translate our findings into an anaesthetic monitoring device, the number of electrodes would need to be reduced to facilitate day-to-day use (e.g., similar to the bispectral index ‘BIS’ monitor). The regional lesioning indicated that the frontal and central regions are important, which is promising for future incorporation into a clinical monitor, as most commonly, only a few electrodes in the frontal region are used clinically. Fourth, we studied exposure to nitrous oxide only. This design allowed us to achieve one of our study aims, which is to isolate the effects of nitrous oxide on EEG recordings. However, to fully understand its translational potential, the model needs to be evaluated on clinical EEG recordings from patients who are administered nitrous oxide in combination with other more potent anaesthetic vapours, which is common clinical practice. Last, in our analysis, we used a combination of manual and automatic artefact rejection techniques before the SVM analysis. This is not possible in a real-time clinical monitor. Hence, the SVM analysis in real-time might be complicated by noise. Comparisons with other machine learning algorithms can include sensitivity to noise analysis.

## Conclusion

This is the first study using a support-vector machine to classify nitrous oxide dosage. We report promising accuracies and generalizability across participants and have identified the delta band in the frontal region as a potentially useful component of multi-channel EEG power spectra. Support-vector classification of nitrous oxide dosage might enable patient monitoring during anaesthesia. This would require further analysis of mixed anaesthetic agent EEG data.

### Supplementary Information

Below is the link to the electronic supplementary material.

Supplementary file1 (DOCX 35 KB)

## Data Availability

The data supporting this study's findings are available from the corresponding author upon reasonable request. EEG analysis code is available online in previous publications.
