# Supplementary material for: Support-vector classification of low-dose nitrous oxide administration with multi-channel EEG power spectra
Source: J Clin Monit Comput. 2023 Jul 13;38(2):363–71. doi: 10.1007/s10877-023-01054-w (PMC10995006; doi:10.1007/s10877-023-01054-w)
Supplement: Supplementary file 1 — Supplementary file1 (DOCX 35 KB) [file 10877_2023_1054_MOESM1_ESM.docx]

Appendix 1 Matlab code

%% SVM modeling

% define which exposures to include in SVM model

classes{1}=[0 40];

classes{2}=[0 20 30 40];

%Frequency lesioning (logical for delta, theta, alpha, beta band)

lesionsgroup{1}=1:10;

lesion{1}=[1 1 1 1]; lesionnames{2}='all';

lesion{2}=[1 0 0 0]; lesionnames{3}='delta';

lesion{3}=[0 1 1 1]; lesionnames{4}='~delta';

lesion{4}=[0 1 0 0]; lesionnames{5}='theta';

lesion{5}=[1 0 1 1]; lesionnames{6}='~theta';

lesion{6}=[0 0 1 0]; lesionnames{7}='alpha';

lesion{7}=[1 1 0 1]; lesionnames{8}='~alpha';

lesion{8}=[0 0 0 1]; lesionnames{9}='beta';

lesion{9}=[1 1 1 0]; lesionnames{10}='~beta';

%Spatial lesioning (logical for regions central(1), left lateral(2), right lateral(3), frontal(4), occipital(5))

electrodegroups=[4,4,2,4,1,2,2,1,1,2,2,5,5,5,5,5,5,5,5,3,3,1,1,3,3,1,4,3,4,4,4,1];

lesionsgroup{2}=[1,2,11:18];

lesion{10}=[1 0 0 0 0]; lesionnames{11}='central';

lesion{11}=[0 1 1 1 1]; lesionnames{12}='~central';

lesion{12}=[0 1 1 0 0]; lesionnames{13}='lateral';

lesion{13}=[1 0 0 1 1]; lesionnames{14}='~lateral';

lesion{14}=[0 0 0 1 0]; lesionnames{15}='frontal';

lesion{15}=[1 1 1 0 1]; lesionnames{16}='~frontal';

lesion{16}=[0 0 0 0 1]; lesionnames{17}='occipital';

lesion{17}=[1 1 1 1 0]; lesionnames{18}='~occipital';

%% shuffle test

n_shuffles=100;

n_participants=numel(unique(Datasettable.participantnr));

confusionmatrix_shuffle=[];acc_shuffle=[];acc_average_shuffle=[];

for i=1:length(classes) % for all classes

n_classes=numel(classes{i});

for repeat = 1:n_shuffles

%create random labels

num=randi([1, n_classes], [n_classes*(n_participants-1), 1]);

random_label=classes{i}(num);

% train and test SVM model

[confusionmatrix_shuffle{i,repeat},acc_shuffle(i,repeat,:),acc_average_shuffle(i,repeat),~]=SVMmodel(Datasettable,classes{i},PredictorNames,random_label);

end

end

acc=[];

acc(:,1,:)=mean(acc_shuffle,2); lesionnames{1}='Shuffle test';

%DIMORD classes,lesions,folds

%% train and test SVM model

confusionmatrix=[];acc_average=[];

for i=1:length(classes) % for all classes

for j=1:length(lesion) % for all lesions

% select PredictorNames based on defined lesion

if length(lesion{j})==4 %frequency lesion

PN_select=PredictorNames(logical(reshape(repmat(lesion{j},numel(channels),1),[],1)));

elseif length(lesion{j})==5 %spatial lesion

PN_select=PredictorNames(repmat(ismember(electrodegroups,find(lesion{j})),1,numel(freqbandnames)));

end

% train and test SVM model

[confusionmatrix{i,j},acc(i,j+1,:),acc_average{i,j},beta]=SVMmodel(Datasettable,classes{i},PN_select);

if (i==1 && j==1)

betas=beta;

end

end

end

%% statistical testing

tests{1}=[1 2];testname{1}='all vs shuffle';

% nonparametric Friedman's test

for i=1:size(acc,1)

for k=1:length(lesionsgroup)

[pF(i,k),tbl,stats] = friedman(squeeze(acc(i,lesionsgroup{k},:))')

criticaldifference(squeeze(acc(i,lesionsgroup{k},:))',lesionnames(lesionsgroup{k}),0.1);

end

for j=1:length(tests)

[p(i,j),h(i,j)]=signrank(squeeze(acc(i,tests{j}(1),:)),squeeze(acc(i,tests{j}(2),:)),'alpha', 0.05/length(tests));

end

end

%% SVM model function

function [confusionmatrix,acc,acc_average]=SVMmodel(Datasettable,classes,PredictorNames,random_label)

%SVMmodel trains and tests a binary of multi-class SVM model with k-folds

%in each fold the data of one participant is hold out of training and used

%as testing data

if nargin>3

shuffle_test=1;

else

shuffle_test=0;

end

for k=1:numel(unique(Datasettable.participantnr)) %fold for each participant

%create training and test dataset

Datasettrain=Datasettable;

Datasettrain(Datasettrain.participantnr==k,:)=[]; %remove data of hold-out participant

if shuffle_test

Datasettrain.dose(find(sum(Datasettrain.dose==classes,2)))=random_label;

end

Datasettest=Datasettable(Datasettable.participantnr==k,:); %data of hold-out participant

%train

if numel(classes)==2 %choose between binary or multi-class model

SVMModel = fitcsvm(Datasettrain,'dose','KernelFunction','linear','ClassNames',classes,'PredictorNames',PredictorNames);

else

t = templateSVM('KernelFunction','linear');

SVMModel = fitcecoc(Datasettrain,'dose','Coding','onevsone','Learners',t,'ClassNames',classes,'PredictorNames',PredictorNames);

end

%test

doses=unique(Datasettest.dose);

indx=find(sum(doses'==classes'));

[predicted_label,~] = predict(SVMModel,Datasettest(indx,PredictorNames));

%calculate confusionmatrix and accuracy

test_y=Datasettest.dose(indx);

confusionmatrix(:,:,k) = confusionmat(test_y,predicted_label);

acc(k)=sum(diag(confusionmatrix(:,:,k)))/sum(sum(confusionmatrix(:,:,k)));

end

acc_average=mean(acc); %average accuracy

end
